# Association of Genetic Polymorphisms of IFNGR1 with the Risk of Pulmonary Tuberculosis in Zahedan, Southeast Iran

**DOI:** 10.1155/2015/292505

**Published:** 2015-11-15

**Authors:** Mohammad Naderi, Mohammad Hashemi, Maryam Rezaei, Abolhassan Safdari

**Affiliations:** ^1^Infectious Diseases and Tropical Medicine Research Center, Zahedan University of Medical Sciences, Zahedan 43181-98167, Iran; ^2^Cellular and Molecular Research Center, Zahedan University of Medical Sciences, Zahedan 43181-98167, Iran; ^3^Department of Clinical Biochemistry, School of Medicine, Zahedan University of Medical Sciences, Zahedan 43181-98167, Iran

## Abstract

*Aim.* The present study was undertaken to find out the possible association between interferon-gamma (IFN-*γ*) receptor 1 (IFNGR1) gene polymorphisms and risk of pulmonary tuberculosis (PTB) in a sample of Iranian population.* Methods*. Polymorphisms of* IFNGR1* rs1327474 (−611 A/G), rs11914 (+189 T/G), rs7749390 (+95 C/T), and rs137854905 (27-bp ins/del) were determined in 173 PTB patients and 164 healthy subjects.* Results*. Our findings showed that rs11914 TG genotypes decreased the risk of PTB in comparison with TT (OR = 0.36, 95% CI = 0.21–0.62, and *p* = 0.0002). The rs11914 G allele decreased the risk of PTB compared with T allele (OR = 0.41, 95% CI = 0.25–0.68, and *p* = 0.0006).* IFNGR1* rs7749390 CT genotype decreased the risk of PTB in comparison with CC genotype (OR = 0.55, 95% CI = 0.32–0.95, and *p* = 0.038). No significant association was found between* IFNGR1* rs1327474 A/G polymorphism and risk/protective of PTB. The rs137854905 (27-bp I/D) variant was not polymorphic in our population.* Conclusion*. Our findings showed that* IFNGR1* rs11914 and rs7749390 variants decreased the risk of PTB susceptibility in our population.

## 1. Introduction

Tuberculosis is an infectious disease and remains a major public health problem as well as leading cause of morbidity and mortality worldwide mostly in Asia and Africa [1, 2]. According to the annual report on global control of TB from WHO, approximately 8.6 million new cases occurred in 2012 [3]. It has been expected that one-third of population is infected with TB, while 5–10% of infected cases will develop active TB during their lifetimes [3], which suggests a role of genetic variation in host immunity. It was expected that the impact of genetic factors on the phenotypic variation and immune responses in the population infected with TB ranges up to 71% [4].

Up to now, we have shown evidence of association between host genetic polymorphisms and PTB susceptibility, including interferon-gamma (IFNG) [5], MIF [6], TLR2 [7], IRGM [8], TIRAP [7], and CD209 [9] in a sample of Iranian population.

IFNG and its receptor (IFNGR1) are key components of innate and adaptive immunity and have been involved in a wide range of infectious and inflammatory disease processes. The IFNG gene has beneficial effects on microbial killing and potentially deleterious consequences [10, 11]. IFNG signaling is mediated through the ligand binding to IFNGR1. Control of IFNGR1 expression level is one of the mechanisms by which cells modulate the potency of IFNG signaling [12]. The receptor for IFNG is made up of *α* and *β* subunits, both of which are integral membrane proteins. The *α* subunit (encoded by the gene IFNGR1) plays a critical role in ligand binding, receptor trafficking, and signal transduction [13]. The human receptor of IFNG (IFNGR) is a heterodimer of IFNGR1 and IFNGR2. The* IFNGR1* gene is located on the long arm of chromosome 6 at position 23.3 (6q23.3), which encodes the ligand binding chain (alpha) of the interferon-gamma receptor. Defects in IFNGR1 have been reported as a cause of Mendelian susceptibility to mycobacterial disease, also known as familial disseminated atypical mycobacterial infection [14].

Several potentially functional single nucleotide polymorphisms (SNPs) have been identified in the human* IFNGR1* gene. Few studies investigated the association between* IFNGR1* gene polymorphisms and risk of TB [15–17]. To the best of our knowledge there is not any report regarding the possible association between IFNGR1 polymorphisms and PTB risk of Iranian population. Thus, the present study aimed to examine the possible associations between polymorphisms of* IFNGR1* gene and susceptibility to PTB in sample of Iranian population.

## 2. Materials and Methods

### 2.1. Study Population

This case-control study was done on 173 PTB patients and 163 healthy subjects. The enrollment process and study design are described elsewhere previously [6, 9, 18]. Briefly, diagnosis of PTB was based on clinical symptoms, radiological evidence, and bacteriological investigations such as sputum Acid Fast Bacillus (AFB) smear positivity, culture, and response to antituberculosis chemotherapy. All control subjects were unrelated adults selected through the population without recent sign, symptom, or history of TB and from the same geographical origin, as the patients with PTB.

The local ethics committee of the Zahedan University of Medical Sciences approved the project, and informed consent was obtained from all individual participants included in the study. Genomic DNA was extracted from the whole blood by the salting out method.

### 2.2. Genotyping

The genotyping of* IFNGR1* rs1327474, rs11914, and rs7749390 polymorphisms was done by PCR-RFLP method. The 27-bp I/D variant was genotyped by PCR methods. The primer sequences with respective annealing temperatures and their amplicon sizes were shown in Table 1.

In each 0.20 mL PCR reaction tube, 1 *μ*L of genomic DNA (~100 ng/mL), 1 *μ*L of each primer, and 10 *μ*L of 2X Prime Taq Premix (Genet Bio, Korea) and the appropriate amount of ddH2O were added.

**Figure 1:**
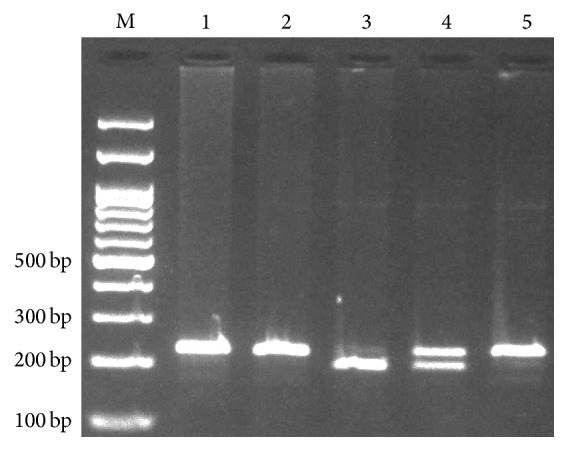
Electrophoresis pattern of polymerase chain reaction restriction fragments length polymorphism (PCR-RFLP) for detection of* IFNGR1* rs1327474 polymorphism. G allele digested by Hpy188I restriction enzyme and produces 204 and 29 bp while A allele undigested (233 bp). M: DNA marker; lanes 1, 2, and 5: AA; lane 3: GG; lane 4: AG.

**Figure 2:**
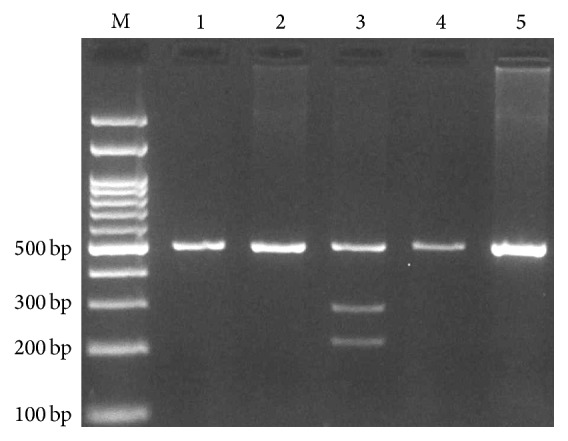
Electrophoresis pattern of PCR-RFLP for detection of* IFNGR1* rs11914 polymorphism. G allele digested by TaqI restriction enzyme and produces 286 and 210 bp while the T allele undigested (496 bp). M: DNA marker; lanes 1, 2, 4, and 5: TT; lane 3: TG.

**Figure 3:**
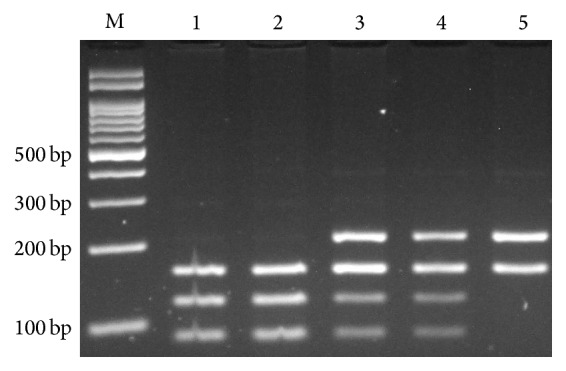
Electrophoresis pattern of PCR-RFLP for detection of* IFNGR1* rs7749390 polymorphism. The 366-bp PCR product digested by BstC8I. C allele produces 87, 121, and 158 bp, while the T allele produces 208 and 158 bp. M: DNA marker; lanes 1 and 2: CC; lanes 3 and 4: CT; Lane 5: TT.

**Figure 4:**
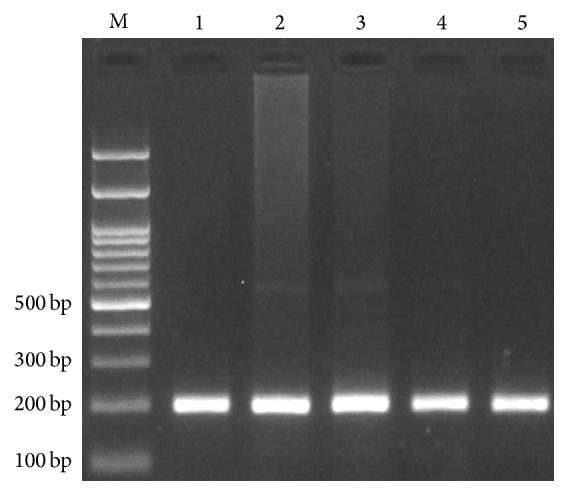
Electrophoresis pattern of PCR for detection of* IFNGR1* rs137854905 (27-bp I/D) polymorphism. I allele produces 223 bp while D allele produces 196 bp. M: DNA marker; lanes 1, 2, 3, 4, and 5: DD.

The PCR cycling conditions for SNPs were the initial denaturation at 95°C for 5 min followed by 30 cycles for 30 s at 95°C and annealing temperature (Table 1) for 30 s and extension at 72°C for 30 s, with a final extension of 72°C for 10 min. The PCR products were verified onto 2.5% agarose gels containing 0.5 *μ*g/mL ethidium bromide and observed under UV light (Figures 1, 2, 3, and 4).

### 2.3. Statistical Analysis

Analysis of the data was done using the SPSS 18.0 software. The differences between the variables were evaluated by chi-square test or independent sample* t*-test according to the data. The associations between genotypes and PTB were calculated by computing the odds ratio (OR) and 95% confidence intervals (95% CI) from logistic regression analyses. *p* value less than 0.05 was considered statistically significant.

## 3. Results

The study consisted of 173 PTB (64 males and 109 females; ages 50.1 ± 20.6 years) patients and 164 healthy subjects (70 males and 94 females; ages 47.5 ± 15.3 years). There was no significant difference between the groups concerning sex and age (*p* > 0.05).

Genotypes and allele frequencies of* IFNGR1* polymorphisms are shown in Table 2.

Our findings indicated that rs11914 TG genotypes decreased the risk of PTB in comparison with TT (OR = 0.36, 95% CI = 0.21–0.62, and *p* = 0.0002). In addition, the rs11914 G allele decreased the risk of PTB in comparison with T allele (OR = 0.41, 95% CI = 0.25–0.68, and *p* = 0.0006). Regarding* IFNGR1* rs7749390 variant, the CT genotype decreased the risk of PTB in comparison with CC genotype (OR = 0.55, 95% CI = 0.32–0.95, and *p* = 0.038).* IFNGR1* rs7749390 T allele was not associated with PTB risk.

No significant association was observed between* IFNGR1* rs1327474 polymorphism and risk/protective of PTB. We found that rs137854905 (27-bp I/D) variant was not polymorphic in our population.

## 4. Discussion

The clinical outcome of TB infection varies noticeably among individuals. Host immunity and genetic factors may be involved in susceptibility or resistance to TB. It is believed generally that IFNG plays a key role in the pathogenesis of tuberculosis [19]. Individuals with inherited disorders of IFNG mediated immunity appear to be specifically vulnerable to TB infections [20]. It has been proposed that IFNG can activate murine macrophages to inhibit* M. tuberculosis* growth [21]. During TB infection, IFNG is upregulated and secreted as a main cytokine to activate macrophages [22]. Defects in human IFNGR1 have been shown to be associated with dominant susceptibility to mycobacterial infection [14]. Our previous study indicated an association between* IFNG* +874 T/A polymorphism and susceptibility to PTB [5]. In the present study we examined the possible association between* IFNGR1* gene polymorphisms and the risk of PTB in a sample of Iranian population. We found that rs11914 TG genotype as well as G allele significantly decreased the risk of PTB. Concerning* IFNGR1* rs7749390 polymorphism, we found that CT genotype decreased the risk of PTB in comparison with CC genotype. The rs1327474 A/G as well as rs137854905 (27-bp I/D) variant of* IFNGR1* polymorphism was not associated with risk/protective of PTB (*p* > 0.05).

Lü et al. [16] showed that rs2234711, rs1327475, and rs7749390 polymorphisms of* IFNGR1* gene were significantly associated with the altered risks of TB. The rs2234711 and rs7749390 polymorphisms decreased the risk of TB, while the rs1327475 variant increased the risk of PTB. They found no significant association among rs3799488 and rs9376267 polymorphisms and risk of PTB.

Bulat-Kardum et al. [15] investigated* IFNGR1* −611 G/A and −56 T/C gene promoter polymorphisms in TB patients and found no significant association between the variants and TB risk in Croatian population. He et al. [17] have found a significant association between* IFNGR1* rs7749390 polymorphism and TB in Chinese Han population.

The discrepancy between studies regarding the impact of* IFNGR1* polymorphisms on PTB risk may be due to the phenotype definition both in cases and in controls, population genetic factors, and even potential global differences in* M. tuberculosis* strain.

In summary, our finding emphasizes the impact of* IFNGR1* polymorphisms on PTB risk in a sample of Iranian population. Further studies with larger sample sizes and different ethnicities are warranted to confirm these findings.

## Figures and Tables

**Table 1:** Primers sequences used for detection of *INFGR1 *gene polymorphisms.

* INFGR1 *polymorphisms	Sequence (5′→3′)	Restriction enzyme	Product size (bp)	Annealing temperature (°C)
rs1327474 (−611 A/G)	F: AGAGCAGACCTCTTCATGAGAGGCTGTCT	Hpy188I	G allele: 204, 29 A allele: 233	63
	R: ACATTTTTAGAAGAGAATGAGACTTCAAA			

rs11914 (+189 T/G)	F: GCCATTTGGTGGTCCATTAC	TaqI	G allele: 286, 210 T allele: 496	64
	R: TCCAGACAGCTGGAATCAGT			

rs7749390 (+95 C/T)	F: CTCTTTCTCCTACCCCTTGTCAT	BstC8I	C allele: 87, 121, and 158 T allele: 208, 158	62
	R: CAGCGCATAATCGTATTTAAAAGTG			

rs137854905 (27-bp I/D)	F: GCAGCCATCTGACTCCAATAG	—	I allele: 223 D allele: 196	67
	R: TTTGGGGGAAATTCTGAGTC			

**Table 2:** Frequency distribution of *INFGR1* gene polymorphisms in PTB and control subjects.

Gene polymorphisms	PTB *n* (%)	Controls *n* (%)	OR (95% CI)	*p*
rs1327474 (−611 A/G)				
AA	56 (32.4)	63 (38.4)	1.00	—
AG	105 (60.7)	94 (57.3)	1.26 (0.79–1.98)	0.354
GG	12 (6.9)	7 (4.3)	1.93 (0.71–5.24)	0.223
Allele				
A	217 (62.7)	220 (67.1)	1.00	—
G	129 (37.3)	108 (32.9)	1.21 (0.88–1.66)	0.258
rs11914 (+189 T/G)				
TT	148 (85.5)	112 (68.3)	1.0	—
TG	25 (14.5)	52 (31.7)	0.36 (0.21–0.62)	0.0002
GG	0 (0.0)	0 (0.0)	—	—
Allele				
T	321 (92.8)	276 (84.1)	1.00	—
G	25 (7.2)	52 (15.9)	0.41 (0.25–0.68)	0.0006
rs7749390 (+95 C/T)				
CC	48 (27.7)	34 (20.7)	1.00	—
CT	63 (36.4)	81 (49.4)	0.55 (0.32–0.95)	0.038
TT	62 (35.8)	49 (29.9)	0.88 (0.49–1.57)	0.768
Allele				
C	159 (45.9)	149 (45.4)	1.00	—
T	187 (54.1)	179 (54.6)	0.98 (0.72–1.33)	0.938
rs137854905 (27-bp I/D)				
DD	173 (100)	164 (100)	—	—
ID	0 (0.0)	0 (0.0)	—	—
II	0 (0.0)	0 (0.0)	—	—
Allele				
D	346 (100)	328 (100)	—	—
I	0 (0.0)	0 (0.0)	—	—
